# Fluid shear stress induced-endothelial phenotypic transition contributes to cerebral ischemia–reperfusion injury and repair

**DOI:** 10.1063/5.0174825

**Published:** 2024-02-26

**Authors:** Denglian Sun, Jia Ma, Lingyu Du, Qiao Liu, Hongyan Yue, Chengxiu Peng, Hanxiao Chen, Guixue Wang, Xiaoheng Liu, Yang Shen

**Affiliations:** 1 Institute of Biomedical Engineering, West China School of Basic Medical Sciences and Forensic Medicine, Sichuan University, Chengdu 610041, China; 2 Jinfeng Laboratory, Chongqing 401329, China; 3 Key Laboratory of Biorheological Science and Technology of Ministry of Education, State and Local Joint Engineering Laboratory for Vascular Implants, College of Bioengineering, Chongqing University, Chongqing 400030, China

## Abstract

Long-term ischemia leads to insufficient cerebral microvascular perfusion and dysfunction. Reperfusion restores physiological fluid shear stress (FSS) but leads to serious injury. The mechanism underlying FSS-induced endothelial injury in ischemia–reperfusion injury (IRI) remains poorly understood. In this study, a rat model of middle cerebral artery occlusion was constructed to explore cerebrovascular endothelial function and inflammation *in vivo*. Additionally, the rat brain microvascular endothelial cells (rBMECs) were exposed to a laminar FSS of 0.5 dyn/cm^2^ for 6 h and subsequently restored to physiological fluid shear stress level (2 dyn/cm^2^) for 2 and 12 h, respectively. We found that reperfusion induced endothelial-to-mesenchymal transition (EndMT) in endothelial cells, leading to serious blood–brain barrier dysfunction and endothelial inflammation, accompanied by the nuclear accumulation of Yes-associated protein (YAP). During the later stage of reperfusion, cerebral endothelium was restored to the endothelial phenotype with a distinct change in mesenchymal-to-endothelial transition (MEndT), while YAP was translocated and phosphorylated in the cytoplasm. Knockdown of YAP or inhibition of actin polymerization markedly impaired the EndMT in rBMECs. These findings suggest that ischemia–reperfusion increased intensity of FSS triggered an EndMT process and, thus, led to endothelial inflammation and tissue injury, whereas continuous FSS induced a time-dependent reversal MEndT event contributing to the endothelial repair. This study provides valuable insight for therapeutic strategies targeting IRI.

## I. INTRODUCTION

As one of the leading causes of death globally, ischemic stroke seriously threatens human health and has aroused much attention.^1^ Currently, the primary clinical treatment approaches involve drug thrombolysis and surgical thrombectomies. However, rapid reperfusion following these procedures may potentially impair cerebral function and deteriorate the prognosis of the stroke, resulting in cerebral ischemia–reperfusion injury (IRI). IRI is a complex multifactorial process, including inflammation, oxidative stress, and leukocyte infiltration, which ultimately leads to neurological dysfunction.^2^

It is well-known that hemodynamic changes play a major role in the process of IRI, recognized as the main cause of endothelial dysfunction and tissue damage. Fluid shear stress (FSS), which is generated by the directional shearing of blood flow, exerts direct effects on cerebral microvascular endothelial cells.^3^ As the main regulator of vascular homeostasis, vascular endothelial cells respond to FSS by a variety of mechanosensory complexes, converting mechanical stimuli into biochemical signals.^4^ Normal physiological fluid shear stress (PFSS) plays a crucial role in maintaining morphology and physiological function of endothelial cells. However, ischemic stroke leads to a decrease in cerebral blood flow, causing endothelial cells to be exposed to relatively low shear stress (LSS). Interestingly, reperfusion therapy restores PFSS, but it also increases the risk of more severe reperfusion injury. Of note, serious IRI and brain tissue damage may occur once the FSS restores from low intensity to normal conditions. Nevertheless, the mechanism by which FSS regulates endothelial function in reperfusion injury and repair remains elusive.

Endothelial–mesenchymal transformation (EndMT) is a complex biological process in which endothelial cells lose their endothelial properties but gain a mesenchymal phenotype, accompanied by the acquisition of cellular motility and contractile properties, elevated expression of mesenchymal markers (N-cadherin, Snail, Slug, α-SMA, etc.).^5,6^ Numerous external biochemical and biophysical stimuli, including transforming growth factor-β (TGF-β), and mechanical stress can stimulate the occurrence of EndMT in vascular endothelial cells.^7^ Mechanical stress acts as a trigger during atherosclerosis and vascular injuries. Oscillatory shear stress (OSS) and LSS can accelerate the formation of human atherosclerotic plaques, while high-level laminar shear stress exhibits resistance against atherosclerosis.^8,9^ Extensive research has elucidated the significant role of fluid dynamics in driving EndMT in atherosclerosis. Recent studies have shown that endothelial cells are capable of undergoing EndMT, subsequently leading to endothelial inflammation and dysfunction, which plays an important role in the development of vascular stenosis and remodeling.^10^ Although previous studies have demonstrated the contribution of EndMT to atherosclerotic lesions and plaque instability,^11^ the effects of FSS-induced EndMT on the cerebral IRI have not been fully elucidated. Contrary to EndMT, mesenchymal–endothelial transformation (MEndT) represents a reverse biological transition from a mesenchymal to an endothelial cell phenotype, which restores endothelial cell properties and functions through the up-regulation of endothelial-related markers. Clinical investigations have revealed that the process can benefit vascular proliferation and improve cardiac function. It is indicated that MEndT contributed to cardiac neovascularization.^12^ Recently, Chen *et al.*^13^ demonstrated that IRI of the brain induces EndMT and vascular fibrosis, suggesting that EndMT has great potential for the treatment of cerebral vascular fibrosis. However, there is currently no direct evidence that the changes in the mechanical environment induced the EndMT during reperfusion. Therefore, we speculated that decreased FSS triggered an EndMT of endothelial cells in early reperfusion, whereas endothelial cells adapted to continuous PFSS and experienced a reversal MEndT to regain their endothelial phenotype.

Endothelial inflammation is closely related to the occurrence and development of EndMT.^5^ Studies have shown that inflammation is a prevalent pathological characteristic of reperfusion injury and plays an important role in secondary brain injury.^14^ Once the vascular blood supply is blocked under stroke conditions, endothelial cells can sensitively respond to the changed FSS and blood-derived signals,^15^ followed by producing adhesion factors (ICAM-1, VCAM-1, etc.) and recruiting leukocytes (neutrophils, monocytes cells, and lymphocytes, etc.) to initiate inflammatory responses.^16^ However, following reperfusion therapy, ischemic brain tissue occurred with more severe inflammatory damage responses due to the restoration of local physiological blood flow. The aggravation of this inflammatory injury is closely associated with endothelial cell dysfunction.^17^ However, further investigation is required to explore the relationship between endothelial inflammation and EndMT in endothelial cells during cerebral ischemia–reperfusion injury and repair.

As a well-known mechanotransducer, Yes-associated protein (YAP) and transcriptional coactivator with PDZ-binding motif (TAZ) respond to mechanical stimuli and translocate into the nucleus to initiate the transcription of genes. Our previous works indicated that FSS induces translocation of YAP from the cytomembrane to the nucleus, promoting epithelial–mesenchymal transition (EMT), and enhancing metastasis in hepatocellular carcinoma.^18^ Studies have revealed that different fluid patterns, such as turbulent or laminar flow and shear stress, are important regulatory factors for inducing cytoskeleton rearrangement and triggering YAP activation in the nucleus.^19^ It has also been demonstrated that flow-dependent YAP/TAZ activities regulate endothelial phenotypes in atherosclerosis,^20^ and YAP serves as mechanoresponsive molecules to regulate endothelial inflammation.^21^ In response to extracellular mechanical signals, Rho GTPases (RhoA, Rac1, Cdc42) could control the nuclear translocation of YAP by promoting actin polymerization and stress fiber formation.^22^ The rearrangement of F-actin inhibits LATS1/2 activity and facilitates the transcription of YAP within the nucleus through both Hippo-dependent and Hippo-independent pathways.^23^ As a key transcription factor to initiate EMT, Snail forms a complex with YAP/TAZ to activate downstream TEAD and Runx2 target genes.^24^ However, it remains unclear whether YAP plays its transcriptional role in regulating EndMT and endothelial inflammatory responses during cerebral ischemia–reperfusion injury and repair.

In this study, an *in vivo* model of middle cerebral artery occlusion [MCAO, Fig. S1(a)] and an *in vitro* parallel plate flow chamber model [Fig. S1(b)] were constructed to explore the effect of FSS on endothelial phenotype transition and inflammation during the process of IRI. Furthermore, using the mRNA sequencing technique, we focused on cytoskeleton-related signaling pathways that triggered the activation of YAP. Our findings provide a novel perspective for the therapy of IRI.

**FIG. 1.**
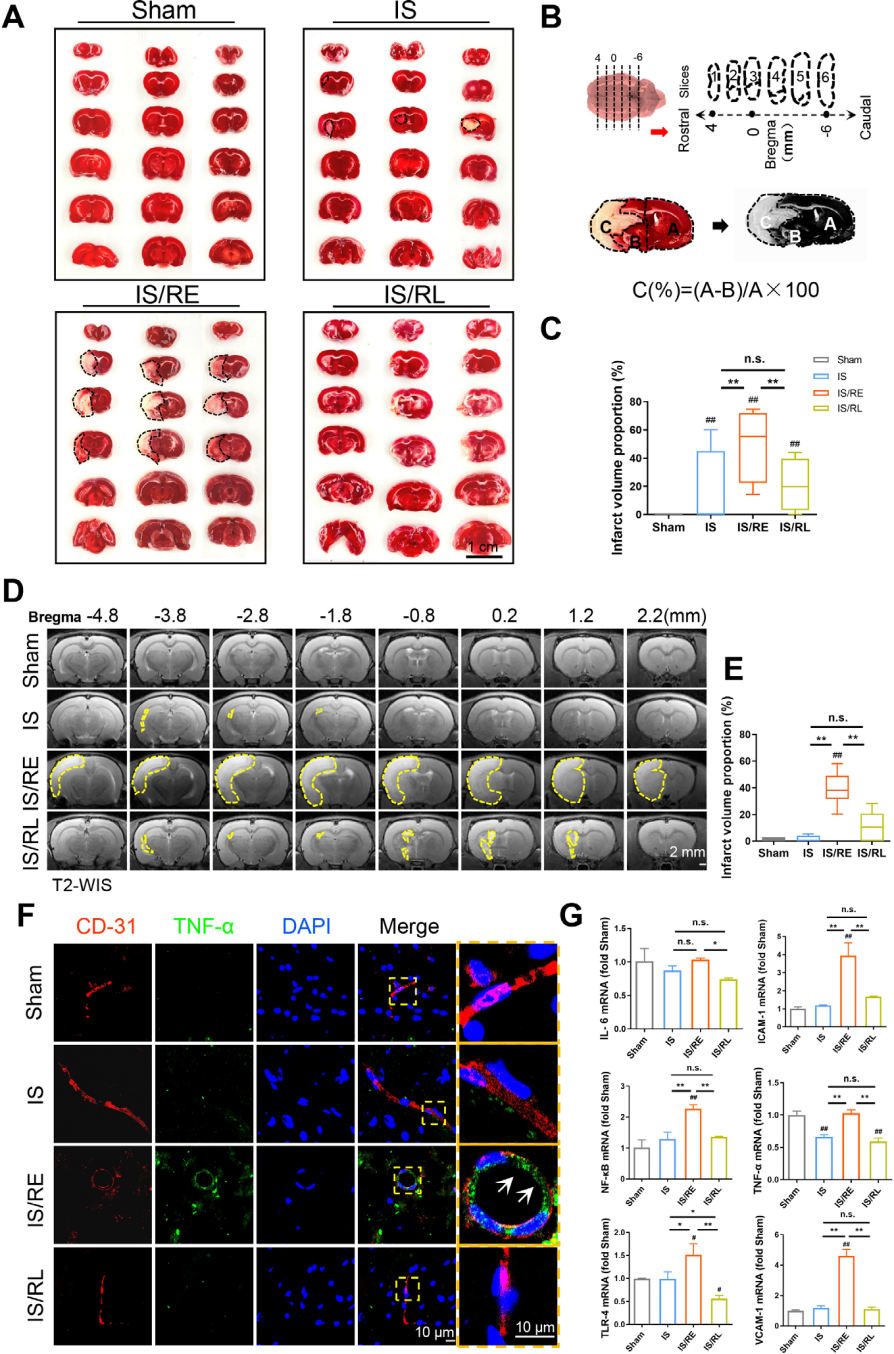
Brain damage and inflammatory response during ischemia–reperfusion. (a) Representative photographs of TTC-stained coronal brain sections before and after ischemia–reperfusion. Red: normal brain tissue. White: infarction tissue. Black dotted line: infarcted area, scale bar = 1 cm; (b) schematic diagram of the location of brain tissue sections and infarction volume calculation method: C(%) = (A − B)/A × 100 [(a): right normal tissue volume and (b): left normal tissue volume], to reduce the effect of edema on the infarct volume. (c) Statistics of infarction rate corresponding to (a). n = 3. (d) Selected coronal MRI images acquired Sham, IS, IS/RE, and IS/RL groups, showing focal lesions in T2-weighted images in different brain areas. Black: normal brain tissue. Gray: infarction tissue. Yellow dotted line: infarct area, scale bar = 2 mm; (e) the statistics of the infarction rate in (d). (f) Immunofluorescence of TNF-α in the cerebral microvascular endothelial cell from sham, IS, IS/RE, and IS/RL subjects were performed. Antibodies against CD31 (red) and TNF-α (green) were used. Nuclei were stained with DAPI (blue). White arrows show TNF-α in the cerebral microvascular endothelial cell. Scale bar = 10 *μ*m (designated regions indicated by yellow square frames were enlarged to show the details.); (g) mRNA expression of inflammation-related factors (IL-6, ICAM-1, VCAM-1, NF-ĸB, TNF-α, and TLR-4) in the cerebral microvascular endothelial cell were measured by qRT-PCR. ^*^ indicates groups comparisons between the IS, IS/RE, and IS/RL groups. # indicates the IS groups, IS/RE groups, and IS/RL groups, respectively, compared to the Sham groups. ^*^ and #, indicate *P* < 0.05 denoted the statistical differences are significant; ^**^ and ## indicate *P* < 0.01 denoted the statistical differences are high significance. Data were shown as means ± standard deviation (SD) based on three different experiments (n = 3).

## II. RESULTS

### A. Endothelial inflammation increased at the early stage of reperfusion

The laser speckle was used to record CBF levels before surgery (baseline) and after reperfusion, respectively. As shown in Fig. S2(a), the blood flow in the rat midbrain was considerably reduced when inserting nylon thread into the left middle cerebral artery for 2 h and returned to physiological level when nylon thread was removed for 10 min, as the blood flow returned from about 200 to 400 [Fig. S2(b)]. It could be found that the cerebral blood flow was successfully blocked to simulate the ischemia period, and the CBF index approached 0.4 compared to the baseline (*P* < 0.001) [Fig. S2(c)]. In addition, it was resumed to simulate the early stage of reperfusion when the nylon thread was removed and the CBF index approached 0.8 [Fig. S2(c)]. All results suggested that the MCAO model was successfully constructed.

Subsequently, the TTC staining of coronal sections of the MCAO model was used to evaluate the infarct volume [Figs. 1(a) and 1(b)]. The statistical results indicated that treatment with reperfusion for 1d (IS/RE groups) after ischemia markedly increased brain infarct sizes. However, reperfusion for 30d (IS/RL groups) was merely a little infarct volume [Fig. 1(c)]. Consistent with the TTC results, magnetic resonance T2 images showed a small infarct volume in the ischemia for 2 h compared to the sham groups. In addition, lesions could be detected in the regions, including periventricular white matter and midbrain in the early stage of reperfusion [Fig. 1(d)], indicating that the infarct volume significantly increased in comparison to the other groups [Fig. 1(e)]. The lesion volume at late reperfusion showed a great recovery of reperfusion injury. Overall, both TTC and MRI results demonstrated that a severe brain injury at the early stage of 1d reperfusion markedly recovered after 30d reperfusion.

We next examined endothelial inflammation at early and late reperfusion. We found that the expression of TNF-α was increased in the endothelial layer after initial reperfusion. Notably, in both IS and IS/RL stages, the expression of TNF-α was significantly lower compared to the IS/RE stage [Fig. 1(f)]. Additionally, the mRNA expression of TLR4, ICAM-1, VCAM-1, IL-6, TNF-α, and NF-ĸB in the cerebral microvascular endothelium was significantly higher in IS/RE stage, compared to IS and IS/RL stage [Fig. 1(g)]. These results strongly indicated that inflammation in the early reperfusion was aggravated significantly but alleviated with an increased duration of reperfusion.

### B. Aggravating inflammation promoted BBB dysfunction

**FIG. 2.**
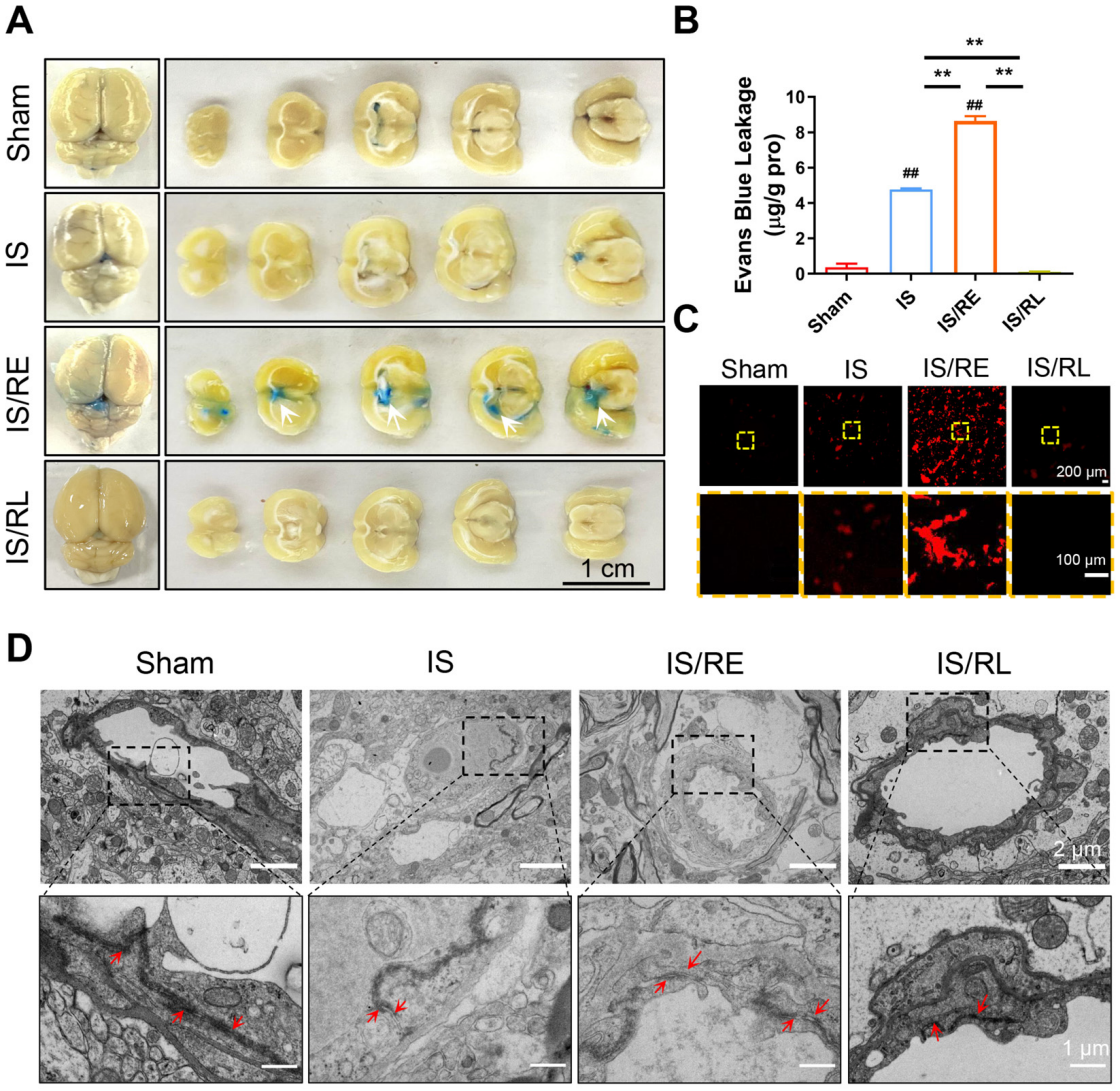
The alterations in the BBB during ischemia–reperfusion. (a) Representative images of the EB-stained brain sections. White arrows show EB's location. Scale bar = 1 cm. (b) Quantification of the EB extravasation. n = 3. (c) Fluorescence intensity of EB extravasation, scale bar =200 *μ*m (designated regions indicated by yellow square frames were enlarged to show the details, scale bar = 100 *μ*m). (d) Typical TEM images of BBB before and after reperfusion (tight junctions were indicated by red arrows). Scale bar = 1 *μ*m, designated regions indicated by black square frames were enlarged to show the details, scale bar = 2 *μ*m. ^*^ indicates groups comparisons between the IS, IS/RE, and IS/RL groups. # indicates the IS groups, IS/RE groups, and IS/RL groups, respectively, compared to the Sham groups. ^*^ and #, indicate *P* < 0.05 denoted the statistical differences are significant; ^**^ and ## indicate *P* < 0.01 denoted the statistical differences are high significance. Data were shown as means ± SD based on three different experiments (n = 3).

Endothelial inflammation increased blood–brain barrier (BBB) permeability has been widely reported.^25,26^ Accordingly, the extravasation of fluorescent EB was quantified at each stage of reperfusion. As shown in Figs. 2(a) and 2(b), the brains in the Sham and IS groups were barely stained with EB. However, it could be found that extravasation of EB was distinctly enhanced in IS/RE groups involving the neocortex and striatum of the left ischemic brain and decreased in IS/RL groups [Fig. 2(c)]. It was suggested that aggravated inflammation increased the permeability of brain barrier under the early stage of reperfusion. We further observed the tight junction of the BBB. The TEM images showed that the structure of the tight junction and brain barrier was lost in the IS/RE groups in comparison to the Sham and IS groups. However, the intact endothelium could be found in IS/RL groups [Fig. 2(d)]. This analysis showed that the aggravation of endothelial inflammation increased BBB permeability at the early reperfusion. The function of the brain barrier was gradually regained with an increased duration of reperfusion.

### C. FSS-induced endothelial inflammation and BBB dysfunction

**FIG. 3.**
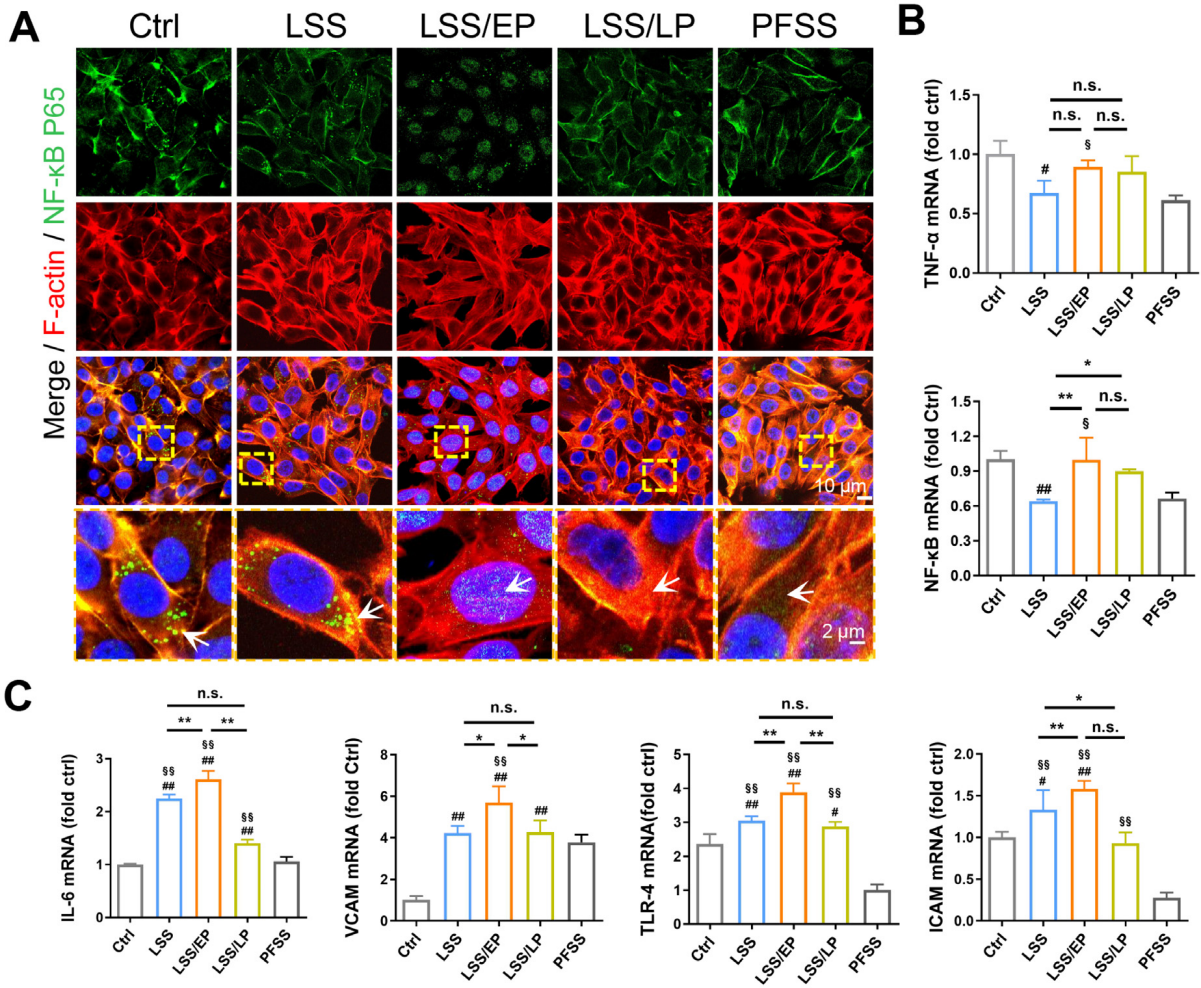

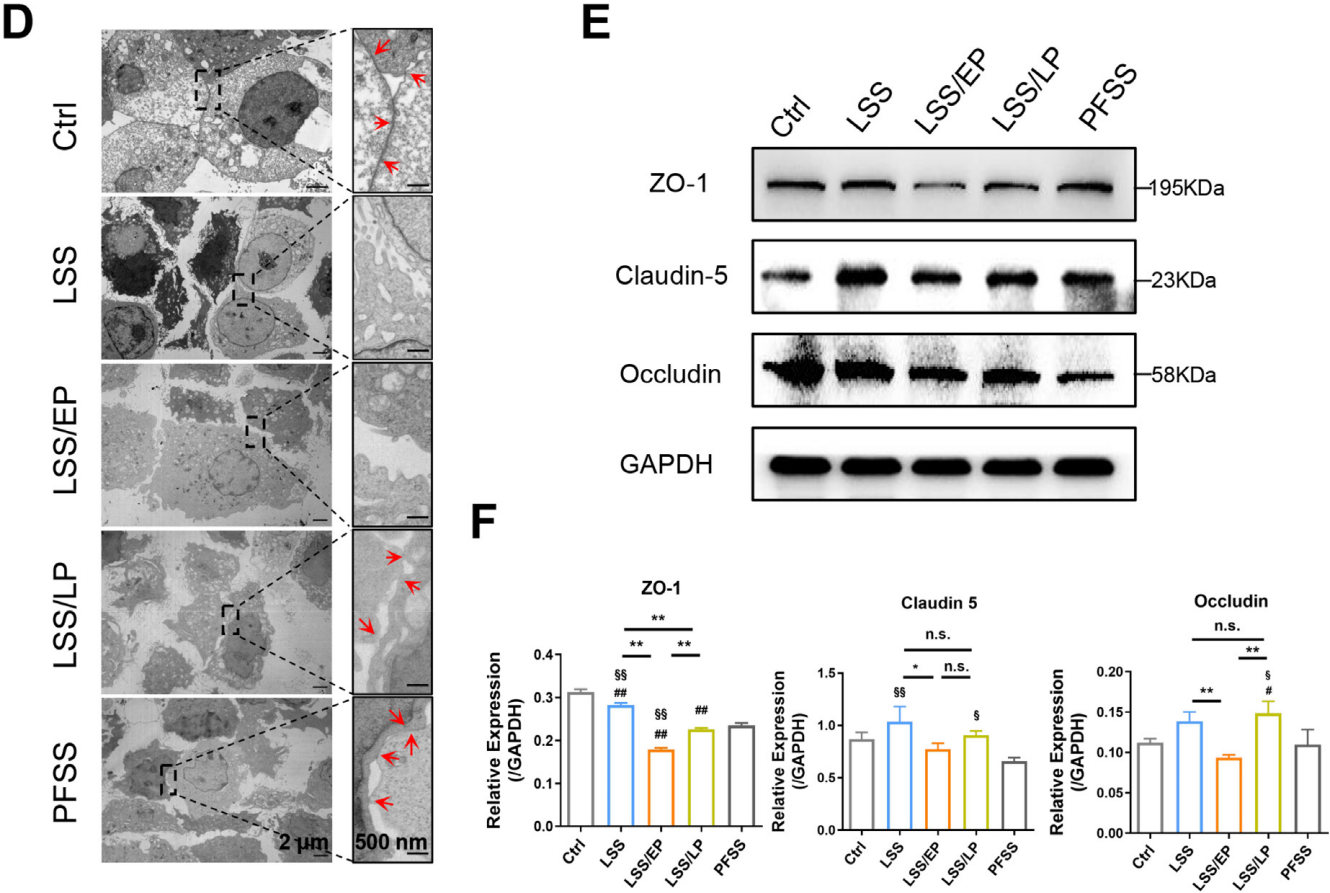
FSS-induced endothelial inflammation and BBB dysfunction. (a) Immunofluorescence images of NF-ĸB P65 (green) and F-actin (red) in rBMECs under different flow conditions. Nuclei were stained with DAPI (blue). The images were captured by confocal microscopes (scale bar = 10 mm). Designated regions indicated by yellow square frames were enlarged to show the details, White arrows show the location of NF-ĸB P65 in rBMECs. Scale bar = 2 *μ*m. (b) and (c) mRNA levels of inflammation-related factors, such as IL-6, ICAM-1, VCAM-1, NF-ĸB, TNF-α, and TLR-4 in the rBMECs, were subject to a different flow. Data were shown as means ± SD. (d) TEM images showed the ultrastructure of the BBB after FSS loading. The red arrows indicated TJs. The scale bar = 2 *μ*m (designated regions indicated by black square frames were enlarged to show the details, scale bar = 500 nm). (e) Representative WB results of TJs-related proteins in rBMECs under different mechanical conditions. (f) Qualifications of Claudin 5, Occludin, and ZO-1 expression in rBMECs after FSS loading. ^*^ indicates groups comparisons between the LSS, LSS/EP, and LSS/LP groups. # indicates the LSS groups, LSS/EP groups, and LSS/LP groups, respectively, compared to the Ctrl groups. ^§^ indicates LSS groups, LSS/EP groups, and LSS/LP groups compared to the PFSS groups, respectively. ^*^, #, and § indicate *P* < 0.05 denoted the statistical differences are significant; ^**^, ##, and §§ indicate *P* < 0.01 denoted the statistical differences are high significance. Data were shown as means ± SD based on three different experiments (n = 3).

The altered mechanical microenvironment in cerebral microvessels is a typical characterization of ischemia–reperfusion. The reduction of blood flow in ischemia restores normal physiological fluid after reperfusion therapy, but more serious reperfusion injury is prone to occur. To further investigate the relationship between FSS and IRI *in vitro*, rBMECs were exposed to 0.5 dyn/cm^2^ of LSS for 6 h (LSS groups) and then increased FSS to 2 dyn/cm^2^ of physiological level for 2 h (LSS/EP groups) and 12 h (LSS/LP groups), respectively. We found that NF-ĸB P65 entered the nucleus from the cytoplasm in the early ischemia–reperfusion stage (LSS/EP groups), which means endothelial inflammation was markedly aggravated [Fig. 3(a)]. Notably, in both LSS and LSS/LP groups, the location of NF-ĸB P65 was in the cytoplasm. Moreover, the mRNA expression of inflammation-related factors, including TLR4, ICAM-1, VCAM-1, IL-6, TNF-α, and NF-ĸB in the LSS/EP groups, were higher than those of LSS and LSS/LP groups [Figs. 3(b) and 3(c)].

However, the LSS/LP groups showed decreased TLR4, ICAM-1, VCAM-1, IL-6, TNF-α, and NF-ĸB expression compared with the LSS/EP groups, suggesting that endothelial inflammation decreased accompanied by the time of reperfusion [Figs. 3(b) and 3(c)]. The above-mentioned results indicated that the early stage of ischemia–reperfusion (exposure to FSS from 0.5 to 2 dyn/cm^2^) induced an aggravation of the inflammatory response. At the same time, with the prolongation of reperfusion time, rBMECs alleviated the inflammatory response.

To further verify the endothelial inflammation-induced dysfunction of BBB, we examined the morphology of TJs by TEM. In control and PFSS groups, cells displayed well-aligned electrodense TJs at the edge of cell borders [red arrows indicated in Fig. 3(d)]. In the LSS groups, under the condition of LSS loading for 6 h, the detached TJs structure could be found in adjacent rBMECs. Especially, the structure of TJs in the LSS/EP groups became irregular, detached, or even lost in some regions, when rBMECs were exposed to 2 dyn/cm^2^ FSS for 2 h [Fig. 3(d)]. Additionally, the expression of TJs-related proteins Claudin 5, Occludin, and ZO-1 of rBMECs in each group were examined. We found that Claudin 5, Occludin, and ZO-1 were significantly reduced in rBMECs in the LSS/EP groups compared to the LSS groups (*P* < 0.05), suggesting that inflammation aggravation induced the TJs loss [Figs. 3(e) and 3(f)]. Consistent with previous results, Claudin 5, Occludin, and ZO-1 were up-regulated in the LSS/LP groups, suggesting that the endothelial barrier recovered with inflammation alleviation under the condition of physiological FSS for 12 h [Figs. 3(e) and 3(f)]. Taken together, these findings demonstrated that increased inflammation led to BBB and endothelial dysfunction through the down-regulation of TJ-related proteins during the early stage of reperfusion. Cells gradually adapted to continuous physiological FSS and restored endothelial phenotype and function.

### D. Analysis of mRNA sequencing

**FIG. 4.**
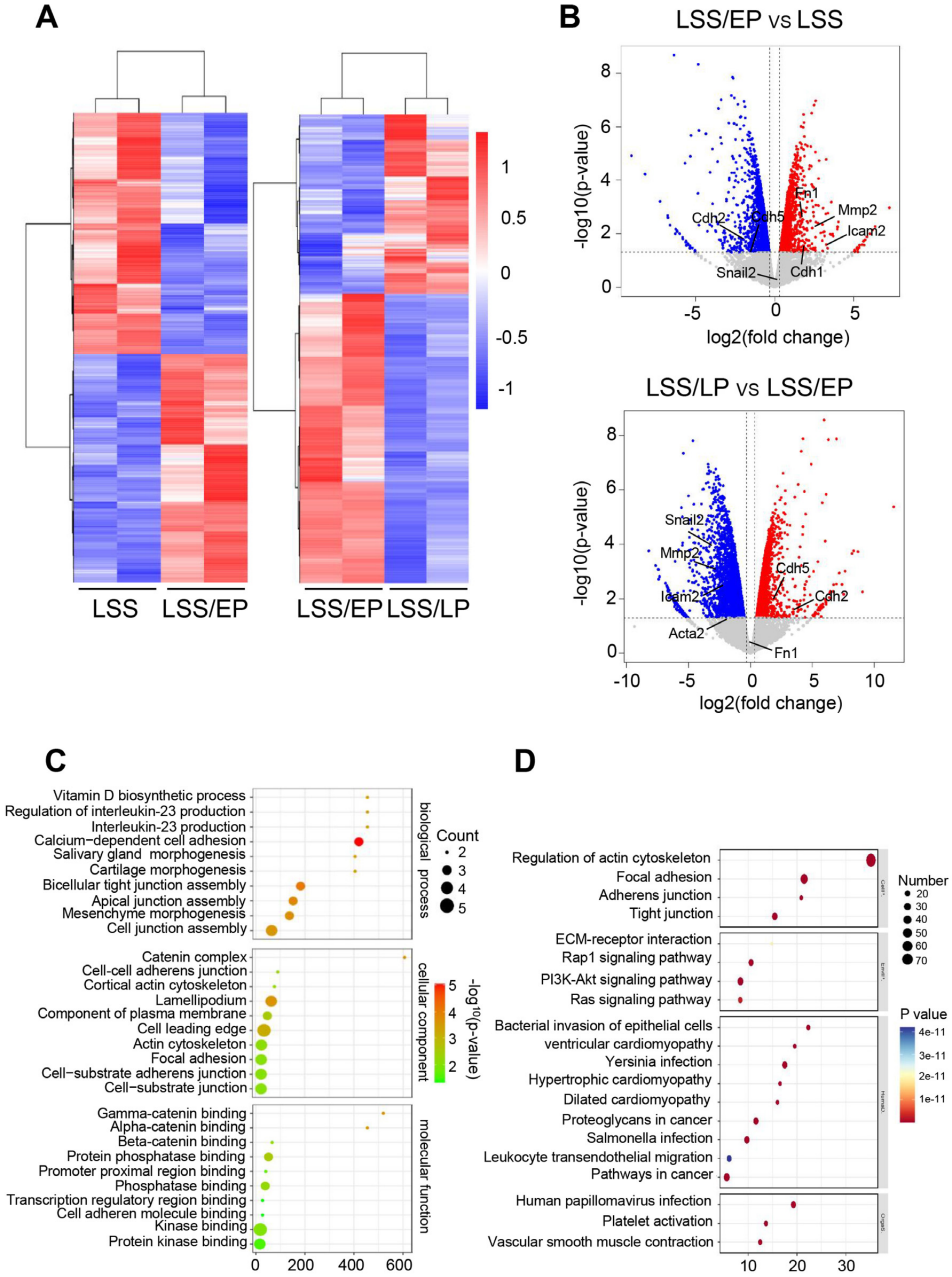
Sequencing of mRNA. (a) Cluster plots of differentially expressed genes in each group. (b) Volcano plots of differentially expressed genes in each group. (c) GO functional cluster gram in each group. (d) KEGG functional cluster gram in each group.

To explore the molecular mechanism of FSS regulation of inflammation of IRI, we performed second-generation mRNA sequencing of rBMECs exposed to FSS, including LSS groups, LSS/EP groups, and LSS/LP groups. Cluster analysis of genes with significant differential expression in each group [Fig. 4(a)]. The clustering of significantly up-regulated genes is denoted by red in the figure, and the clustering of significantly down-regulated genes is denoted by blue. The volcano plot of gene clustering revealed that in the LSS/EP groups compared with the LSS groups, the expression of endothelial marker genes was significantly down-regulated, such as Cdh2 and Cdh5, while the expression of mesenchymal marker genes and inflammation-related genes was significantly up-regulated, like Cdh1, Mmp2, Fn1, and Icam2. However, when the LSS/LP groups were compared with the LSS/EP groups, mesenchymal marker genes, and inflammation-related genes were significantly down-regulated, while endothelial marker genes were significantly up-regulated [Fig. 4(b)].

GO functional enrichment analysis was performed [as shown in Fig. 4(c)]. The results of biological process analysis showed that differential gene expression was highly enriched in biological processes, such as cell junctions, cell adhesion, and mesenchymal morphogenesis. The results of the cellular component analysis revealed that the significantly expressed differential genes mostly originated from cytoskeletal components. Molecular functional analysis showed that the differential gene expression was highly enriched in protein kinase binding, cell adhesion molecule binding, and phosphatase binding functions, and the kinase binding function was particularly significant [Fig. 4(c)].

The KEGG analysis of each group indicated that the main signaling pathways involved in the significantly expressed differential genes were regulation of actin cytoskeleton, focal adhesion, tight junction, PI3K-Akt signaling pathway, and so on. Especially, the actin cytoskeleton pathway showed the most remarkable contribution to IRI [Fig. 4(d)].

### E. Continuously changed FSS induces phenotypic transformation in rBMECs

**FIG. 5.**
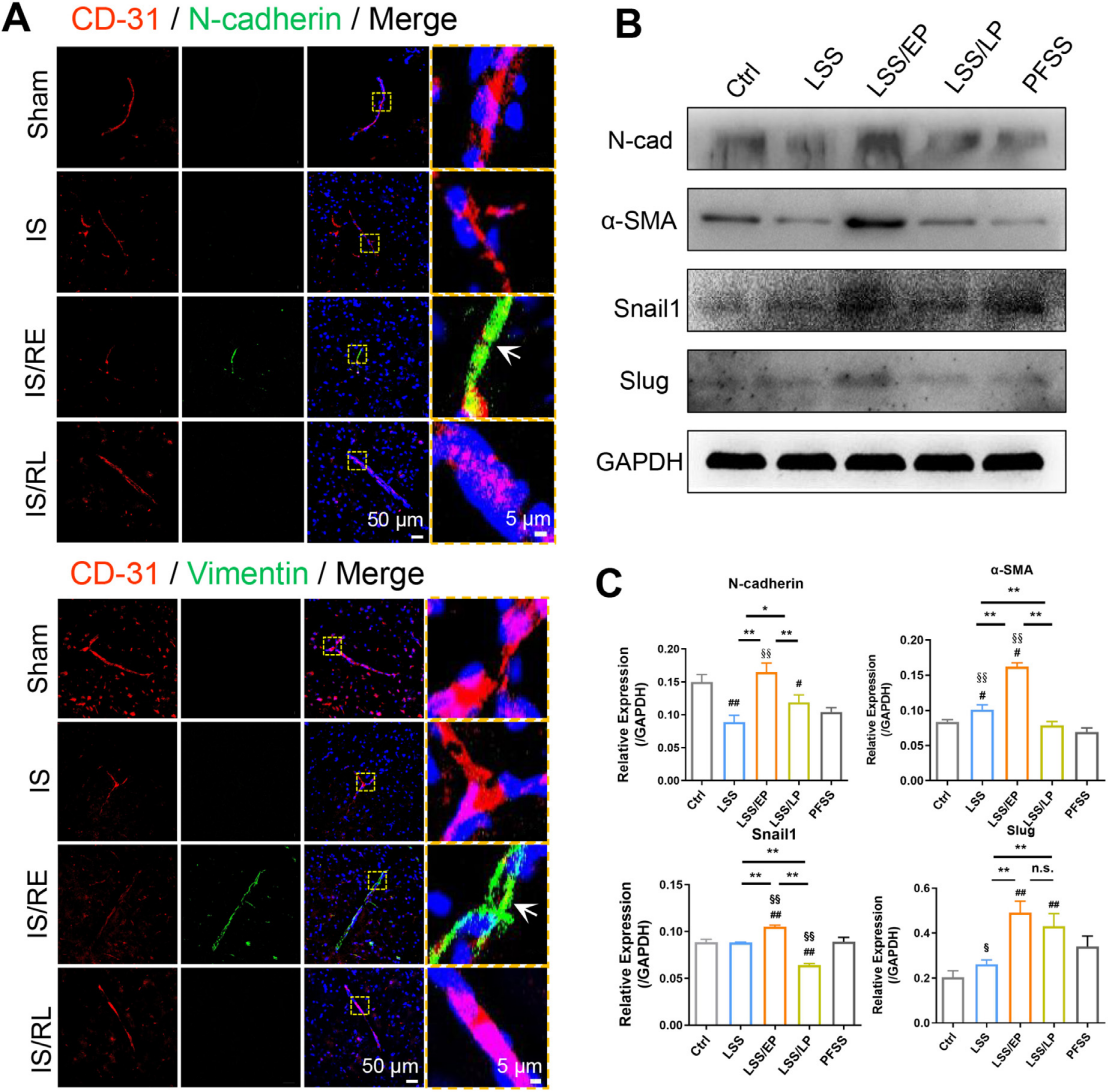

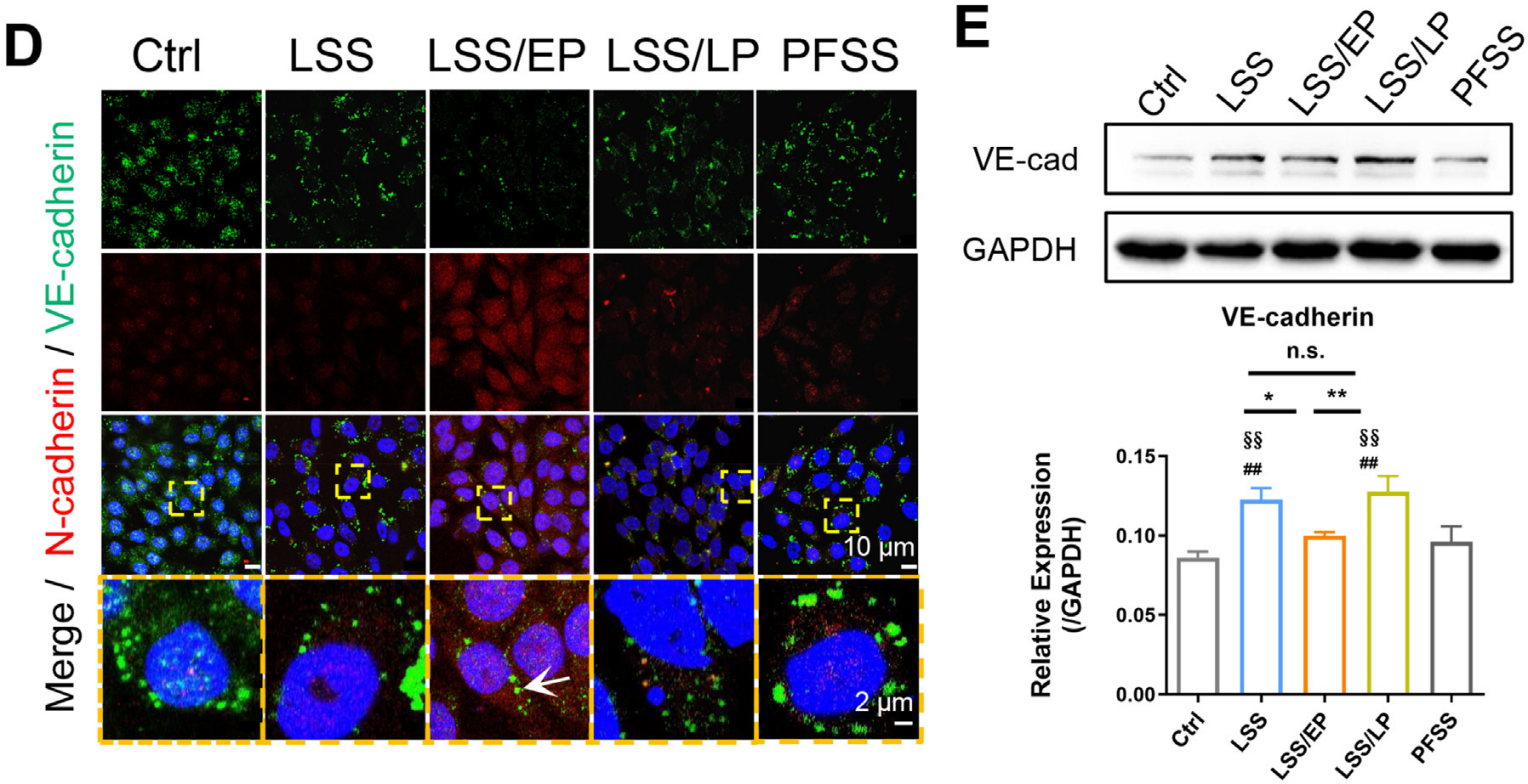
Continuously changed FSS inducing phenotypic transformation in rBMECs. (a) The typical confocal images of double immunofluorescence staining with endothelial marker CD31 (red) and mesenchymal markers N-cadherin, and Vimentin (green); nuclei were labeled with DAPI (blue). Scale bar = 50 *μ*m. Designated regions indicated by yellow square frames were enlarged to show the details, scale bar = 5 *μ*m (n = 3). Western blotting analysis of (b) mesenchymal markers, N-cadherin, Snail1, Slug, and α-SMA, and (e) endothelial marker VE-cadherin in LSS, LSS/EP, and LSS/LP. Quantification of (e) endothelial marker VE-cadherin and (c) N-cadherin, Snail1, Slug, and α-SMA expression level by image analysis of the western blot bands. ^*^ indicates groups comparisons between the LSS, LSS/EP, and LSS/LP groups. # indicates the LSS groups, LSS/EP groups, and LSS/LP groups, respectively, compared to the trl groups. §, indicates LSS groups, LSS/EP groups, and LSS/LP groups compared to the PFSS groups, respectively. ^*^, #, and § indicate *P* < 0.05 denoted the statistical differences are significant; ^**^, ## and §§ indicate *P* < 0.01 denoted the statistical differences are high significance. Data were shown as means ± SD based on three different experiments (n = 3). (d) Typical confocal images of VE-cadherin (red) and N-cadherin (green) in LSS, LSS/EP, and LSS/LP. Nuclei were labeled with DAPI (blue). The white arrows showed the location of VE-cadherin in cells, the scale bar = 10 *μ*m (designated regions indicated by yellow square frames were enlarged to show the details, scale bar = 2 *μ*m).

According to the mRNA Sequencing result, we further explored the mechanism of IRI and repair. We hypothesized that a transition between the endothelial and mesenchymal phenotype of endothelial cells contributed to inflammation in the process of reperfusion. On the one hand, we found that mesenchymal markers (N-cadherin, Vimentin, Snail1, and α-SMA) were markedly up-regulated in vessels in the IS/RE groups compared with the IS groups *in vivo,* suggesting that EndMT occurred in cerebral microvascular endothelial cells. However, these mesenchymal markers were significantly decreased in the IS/RL groups, indicating that a reversed MEndT event occurred in cerebral microvascular endothelial cells [Figs. 5(a) and Figs. S3(a) and S3(b)].

On the other hand, we found that VE-cadherin (endothelial markers) translocated from cytomembrane to cytoplasm in LSS/EP groups [Fig. 5(d)], and its expression level was significantly down-regulated compared to LSS groups *in vitro* [Fig. 5(e)]. While the levels of mesenchymal markers, including N-cadherin, Snail1, Slug, and α-SMA in LSS/EP groups were significantly increased compared to those in LSS groups *in vitro* [Figs. 5(b) and 5(c)]. These results provided strong evidence that an EndMT process was triggered in rBMECs exposed to FSS from 0.5 to 2 dyn/cm^2^. With the increased duration of 2 dyn/cm^2^ from 2 to 12 h, it was interesting that the expression of VE-cadherin significantly up-regulated [Fig. 5(e)], while the level of N-cadherin, Snail1, Slug, and α-SMA in LSS/LP groups showed a sharply decreased, compared with that in LSS/EP groups [Figs. 5(b) and 5(c)]. These results demonstrated that a decreased level of FSS triggered an EndMT process in rBMECs, whereas restored FSS led to a reversal MEndT event in a time-dependent way.

### F. YAP participates in cerebral ischemia–reperfusion injury and repair

**FIG. 6.**
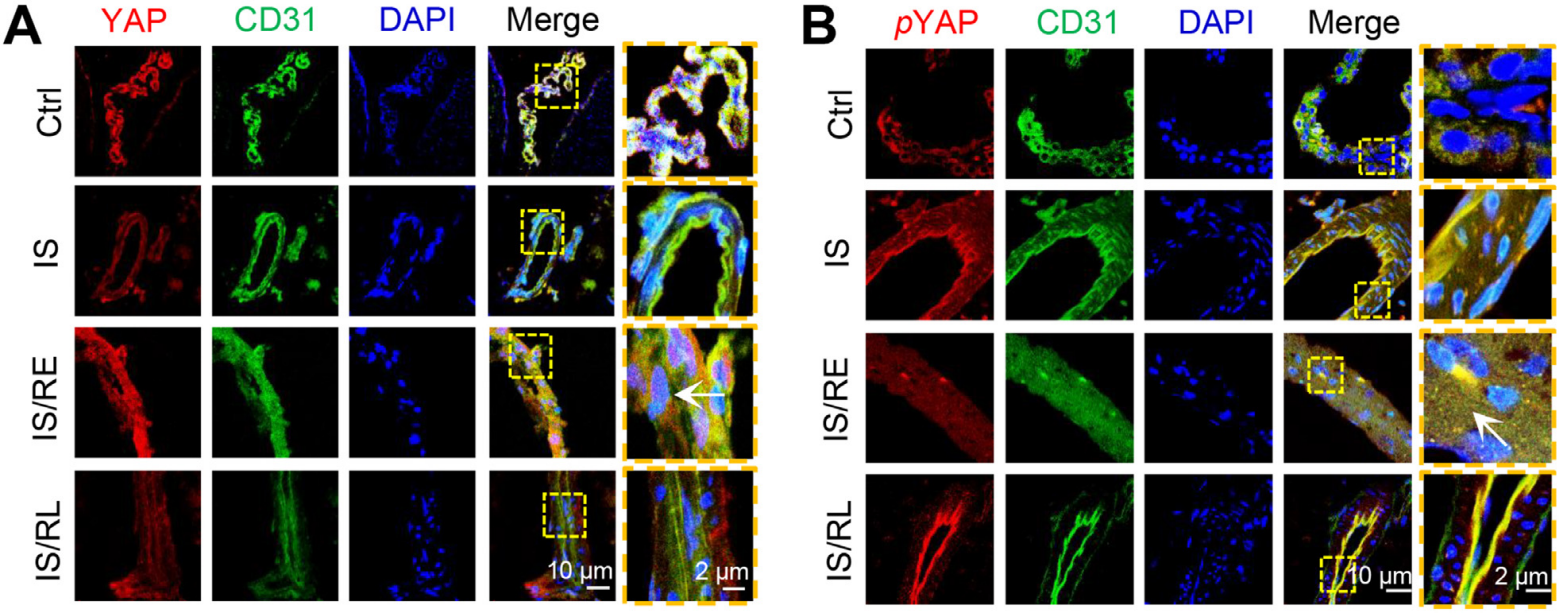

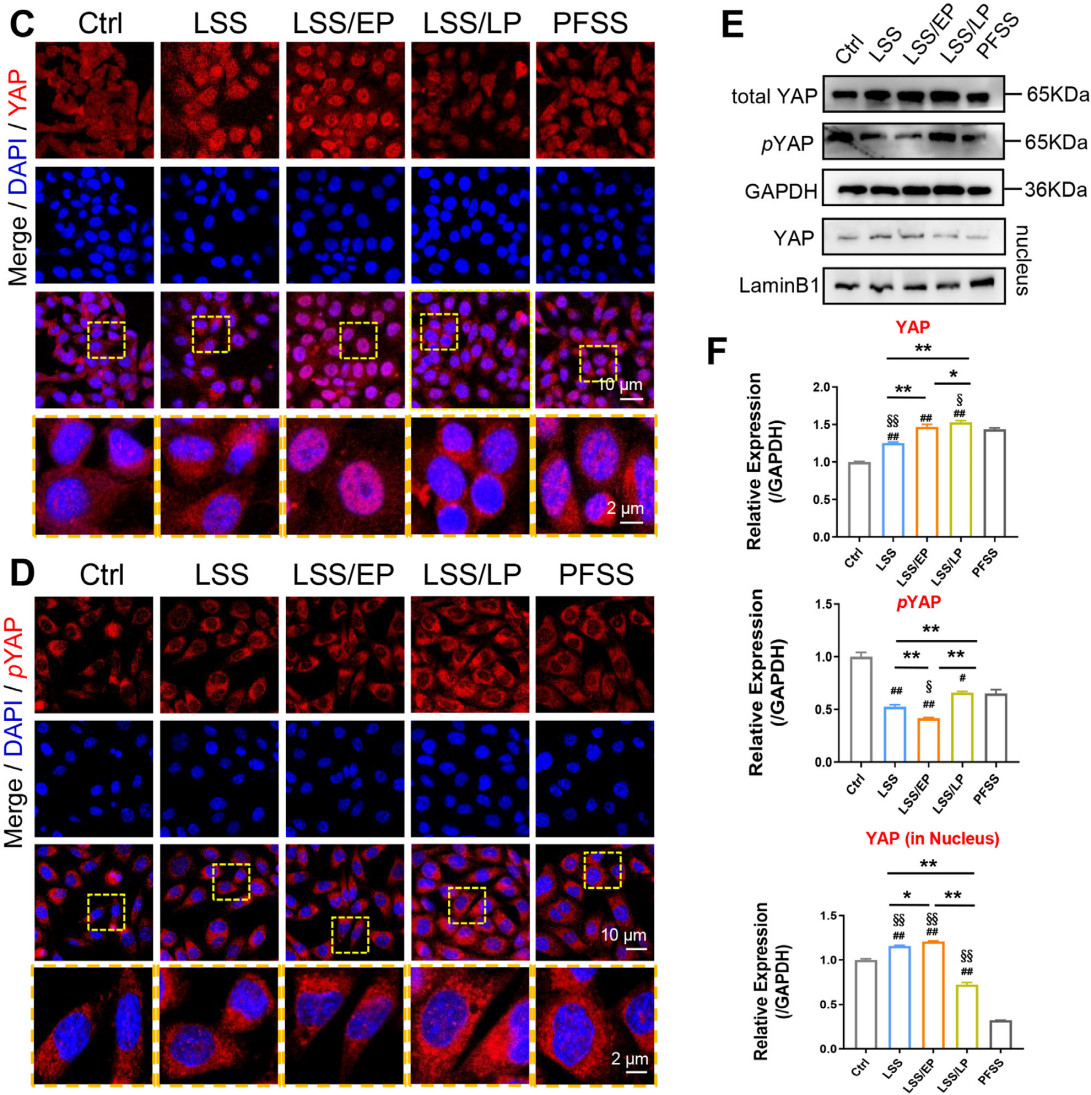
YAP participates in the regulation of cerebral IRI. The typical confocal images of double immunofluorescence staining with endothelial marker CD31 (green) and (a) YAP (red) or (b) *p*YAP (red) after MCAO. Nuclei were labeled with DAPI (blue), scale bar = 10 *μ*m. Designated regions indicated by yellow square frames were enlarged to show the details, and yellow triangles show the nuclear location of YAP in rBMECs, scale bar: 2 *μ*m. The typical immunofluorescence images of (c) YAP (red) or (d) *p*YAP (red) in rBMECs under different flow conditions, scale bar = 10 *μ*m. Designated regions indicated by yellow square frames were enlarged to show the details, scale bar: 2 *μ*m. (e) Western blotting analysis of total YAP, phosphorylated YAP, and nuclear YAP in rBMECs exposed to different flows. (f) Quantification of YAP and *p*YAP expression level by image analysis of the western blot bands. ^*^, indicates groups comparisons between the LSS, LSS/EP, and LSS/LP groups. # indicates the LSS groups, LSS/EP groups, and LSS/LP groups, respectively, compared to the Ctrl groups. §, indicates LSS groups, LSS/EP groups, and LSS/LP groups compared to the PFSS groups, respectively. ^*^, #, and § indicate *P* < 0.05 denoted the statistical differences are significant; ^**^, ## and §§ indicate *P* < 0.01 denoted the statistical differences are high significance. Data were shown as means ± SD based on three different experiments (n = 3).

We next addressed the underlying biomechanical mechanisms by which FSS-induced endothelial cells undergo EndMT and inflammation. YAP was recently shown to be exquisitely sensitive to matrix stiffness and shear stress.^27^ Interestingly, using rat MCAO models, we found that YAP was highly expressed in the IS/RE groups. Compared with the IS groups, the immunostaining indicated that YAP was prominently upregulated and translocated into the nucleus in the IS/RE groups, and markedly expressed in the cytoplasm in the IS/RL groups [Fig. 6(a)]. Phosphorylated YAP (*p*YAP) was expressed in the cytoplasm in all groups. Compared with the IS groups, the level of *p*YAP significantly decreased in the IS/RE groups, but recovered in the IS/RL groups [Fig. 6(b)]. These results suggested that YAP may contribute to the regulation of cerebral ischemia–reperfusion injury and repair.

Using parallel plate flow chambers *in vitro*, the immunostaining also revealed that FSS induced nuclear accumulation [Fig. 6(c)] as well as the expression of *p*YAP was decreased in the LSS/EP groups, compared with the LSS groups [Fig. 6(d)]. The nuclear translocation of YAP and decreased cytoplasmic *p*YAP were also confirmed by western blotting in LSS/EP groups. It could be found that there were significant differences in total YAP levels among the LSS, LSS/EP, and LSS/LP groups. The expression of *p*YAP was significantly down-regulated in the LSS/EP groups but increased in the LSS/LP groups [Figs. 6(e) and 6(f)].

### G. YAP involved in FSS-regulating EndMT

**FIG. 7.**
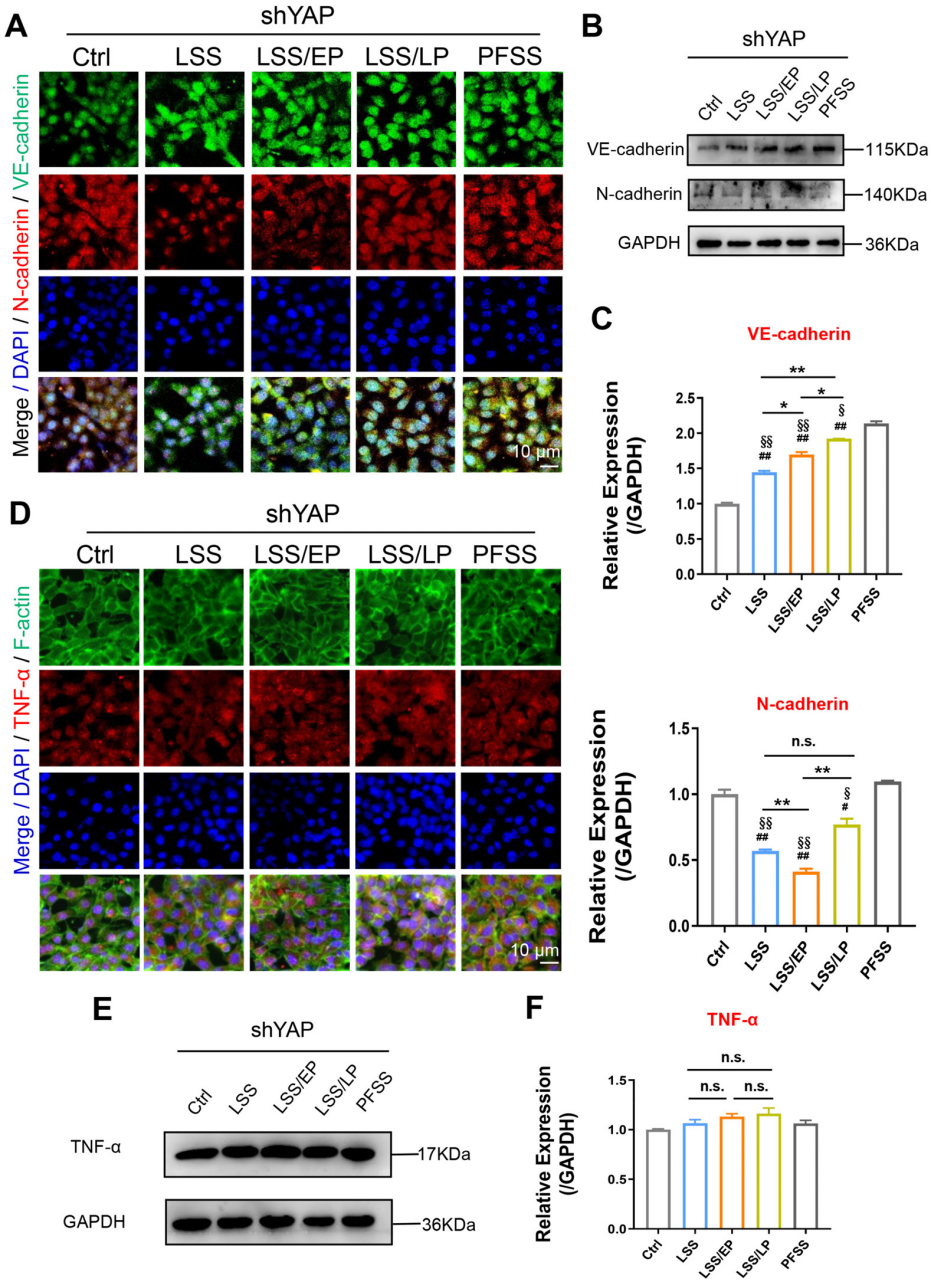
YAP-mediated FSS regulates EndMT in cerebral IRI. (a) The typical confocal images of double immunofluorescence staining with VE-cadherin (green) and N-cadherin (red) in shYAP-rBMECs exposed to different flows. Nuclei were labeled with DAPI (blue). (b) Western blotting analysis of VE-cadherin and N-cadherin in shYAP-rBMECs. Scale bar = 10 *μ*m. (c) Quantification of VE-cadherin and N-cadherin expression level by image analysis of the western blot bands. (d) The distribution of TNF-α (red) in shYAP-rBMECs by immunofluorescence. F-actin was labeled with green fluorescence, and nuclei were labeled with DAPI (blue). The images were captured by confocal microscopes (Scale bar: 10 mm). (e) The expression of TNF-α in shYAP-rBMECs. (f) Quantification of TNF-α expression level by image analysis of the western blot bands. ^*^ indicates groups comparisons between the LSS groups, LSS/EP groups, and LSS/LP groups. # indicates the LSS groups, LSS/EP groups, and LSS/LP groups, respectively, compared to the Ctrl groups. §, indicates LSS groups, LSS/EP groups, and LSS/LP groups compared to the PFSS groups, respectively. ^*^, #, and § indicate *P* < 0.05 denoted the statistical differences are significant; ^**^, ## and §§ indicate *P* < 0.01 denoted the statistical differences are high significance. Data were shown as means ± SD based on three different experiments (n = 3).

To verify the role of YAP in EndMT and inflammation during cerebral IRI, we knocked down YAP by shRNA approach and examined the expression of EndMT markers. shYAP-rBMECs were exposed to FSS (FSS-shYAP) with various durations. In contrast to the previous result in Fig. 5, FSS-shYAP abolished these mesenchymal transitions indicated by upregulation of VE-cadherin and down-regulation of N-cadherin, suggesting that FSS-shYAP rBMECs maintained endothelial phenotype [Figs. 7(a)–7(c)]. It has been reported that the increased expression of MMP-2 is frequently associated with the induction of EMT.^28^ We found that there was no change in MMP2 expression among all groups following YAP knockdown. Furthermore, immunofluorescent and Western blot results revealed an upregulated expression of mesenchymal markers α-SMA in sh-rBMECs exposed to PFSS compared to LSS, but no significant difference was observed between PFSS/EP to PFSS/LP groups [Figs. S4(a)–S4(c)]. Meanwhile, the expression of the inflammatory factor TNF-α showed no significant changes in the LSS, LSS/EP, and LSS/LP phases [Figs. 7(d)–7(f)]. These results further confirmed that YAP was engaged in regulating EndMT, which contributed to cerebral ischemia–perfusion injury and repair.

### H. YAP-mediates EndMT depend on Rho GTPases-F-actin signaling

**FIG. 8.**
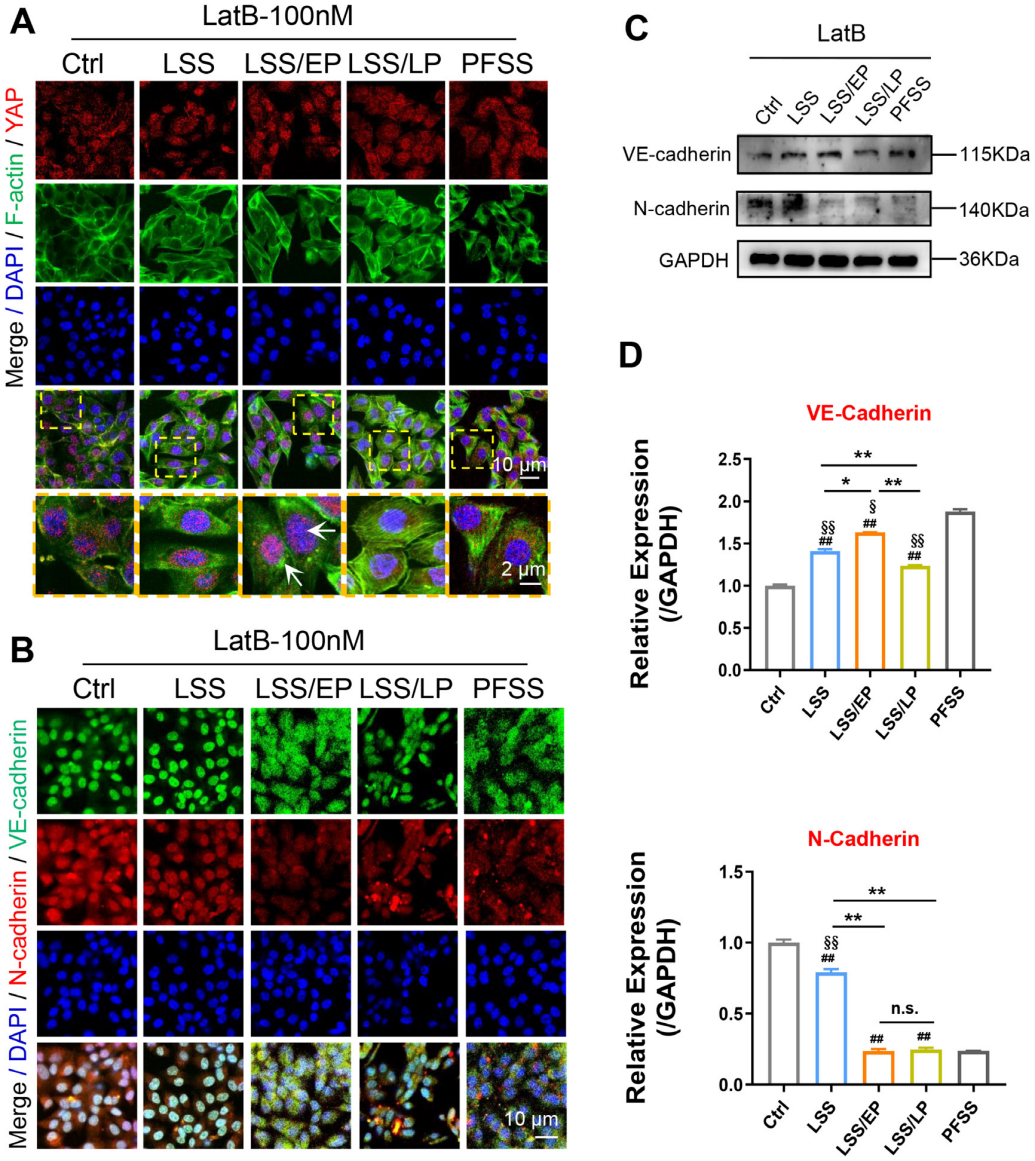
The arrangement of F-actin contributed to FSS-regulating EndMT. The typical confocal images of double immunofluorescence staining with (a) YAP (red) and F-actin (green) and (b) VE-cadherin (green) and N-cadherin (red) in rBMECs after Lat B treatment in LSS, LSS/EP, and LSS/LP. Nuclei were labeled with DAPI (blue). The images were captured by confocal microscopes (Scale bar = 10 mm). The images were captured by confocal microscopes (scale bar = 10 mm). Designated regions indicated by yellow square frames were enlarged to show the details, scale bar = 2 *μ*m. (c) Western blotting analysis of VE-cadherin and N-cadherin in rBMECs after Lat B treatment in LSS, LSS/EP, and LSS/LP. (d) Quantification of VE-cadherin and N-cadherin expression level by image analysis of the western blot bands. ^*^ indicates groups comparisons between the LSS, LSS/EP, and LSS/LP groups. # indicates the LSS groups, LSS/EP groups, and LSS/LP groups, respectively, compared to the Ctrl groups. §, indicates LSS groups, LSS/EP groups, and LSS/LP groups compared to the PFSS groups, respectively. ^*^, #, and § indicate *P* < 0.05 denoted the statistical differences are significant; ^**^, ## and §§ indicate *P* < 0.01 denoted the statistical differences are high significance. Data were shown as means ± SD based on three different experiments (n = 3).

In previous mRNA sequencing analysis, the actin cytoskeleton pathway showed the most remarkable contribution to IRI [Fig. 4(d)]. Mechanical cues modulate the activities of Rho GTPases, which in turn lead to actin cytoskeleton remodeling.^22^ As shown in Figs. S5(a) and S5(b), the results showed that RhoA expression was significantly up-regulated in the LSS groups compared to that in static controls and significantly down-regulated at the stage of LSS/EP. Similarly, the protein levels of Cdc42 [Figs. S5(c) and S5(d)] and Rac1 [Figs. S5(e) and S5(f)] also increased in LSS groups and significantly decreased in LSS/EP groups, suggesting that Rho GTPases responded to FSS and possibly regulated cytoskeletal remodeling. Furthermore, the role of actin in YAP activation was explored. We found that FSS induced remodeling and rearrangement of F-actin fiber, which could be markedly inhibited by cytoskeleton inhibitor Lat B. Moreover, the nuclear accumulation of YAP was attenuated in FSS-HepG2 cells treated with Lat B [Fig. 8(a)]. With the treatment of Lat B, endothelial marker VE-cadherin expression was significantly up-regulated during LSS/EP phase and mesenchymal marker N-cadherin was significantly down-regulated [Figs. 8(b)–8(d)]. Furthermore, no statistically significant differences were observed in the expression levels of MMP2 and α-SMA during the LSS/EP phase [Figs. S4(d)–S4(f)]. Additionally, there was no significant difference found in the expression of inflammatory factor TNF-α among all groups (Fig. S6). This demonstrated that inhibition of the cytoskeleton resulted in rBMECs restoring the endothelial phenotype and reducing inflammation. Taken together, these findings showed that YAP mediated EndMT in cerebral IRI through the Rho GTPases-F-actin signaling pathway.

## III. DISCUSSION

Over the past years, IRI has attracted widespread attention due to its high incidence and risk. A previous clinical study has shown that 6% of patients experienced cerebral hemorrhage after reperfusion therapy,^29^ which can be largely attributed to the sudden alteration in the fluid microenvironment. In this study, we conducted simulations to investigate the fluctuation of FSS during both *in vivo* and *in vitro* ischemia–reperfusion processes, aiming to further elucidate the impact of FSS on cerebrovascular endothelial function.

Existing studies have confirmed that a certain intensity of FSS (2 dyn/cm^2^) is essential for the maintenance of endothelial cell morphology and function under physiological conditions.^30^ In ischemia conditions, the endothelial cells are typically exposed to LSS (0.5 dyn/cm^2^), which can be restored to PFSS following reperfusion therapy. By constructing the MCAO model to simulate ischemia and reperfusion in rat cerebral vessels (Fig. 1), our results intriguingly found that restored FSS led to more severe brain injury and endothelial inflammation in the early stage of reperfusion, which may be alleviated with the increased duration of reperfusion (Fig. 2). Endothelial inflammation has been conceived as an important factor involved in the occurrence of IRI.^31^ It is well known that endothelial cells, lining the blood vessel wall, respond precisely to the action of FSS.^32^ However, the effect of FSS on inflammation is heterogeneous which is closely related to their type, intensity, and duration. Laminar shear stress has anti-inflammatory and anticoagulant effects on endothelial cells,^33^ whereas disturbed fluid shear stress can promote endothelial cell inflammation and endothelial cell response to inflammatory cytokines.^34^ Our previous work also indicated that disturbed flow increased the expression of cathepsin K, which contributed to endothelial inflammation and vascular remodeling, leading to atherogenesis.^35^ In this study, similar to previous *in vivo* results, the *in vitro* results also demonstrated that restored FSS aggravated the occurrence of inflammation in rBMECs at the early ischemia–reperfusion stage (Fig. 3), which may subsequently result in the dysfunction of endothelial cell and brain injury. Interestingly, the IL-6 expression in rBMECs exposed to FSS significantly elevated compared to that observed under static culture conditions [Fig. 3(c)], while we previously discovered a reduction in IL-6 levels in the IS stroke groups, along with BBB dysfunction [Fig. 1(g)]. It has been elucidated that blocking IL-6 suppressed the BBB disorder, and IL-6 regulate morphological and functional changes in BBB.^36,37^ Consequently, the difference in IL-6 response between *in vitro* FFS loading and *in vivo* IS stoke suggests that IL-6 could serve as a selective marker for BBB dysfunction.

Serving as a dynamic interface that maintains the homeostasis of the central nervous system, BBB is mainly composed of cerebral microvascular endothelial cells, pericytes, and astrocyte tail pods.^38^ IRI commonly leads to a breakdown of BBB. Its unique tight and adhesive junctions protect the brain from harmful compounds. Growing evidence identifies that the dysfunction of the BBB and the increase in permeability are the key reasons accounting for the aggravation of brain injury after thrombolytic therapy.^39^ During the ischemic phase, hypoxia leads to cytotoxic edema and BBB dysfunction, whereas during the initial phase of reperfusion, the impairment of BBB is highly associated with inflammation, accompanied by IL-1β and TNF-α production and leukocyte migration.^40^ Consistently, our results identified that aggravated inflammation disrupted the TJs and increased the permeability of the BBB at the early stage of reperfusion. However, the BBB experienced an obvious improvement once the inflammation was reduced (Figs. 2 and 3). Previous studies have shown that in IRI, cerebrovascular endothelial cells undergo a stress response that activates NF-ĸB, thereby promoting the secretion of pro-inflammatory cytokines, including interleukin-8 (IL-8), TNF-α, MCP-1, interferon γ (IFN-γ), and chemotaxis.^41^ This is followed by the release of neutrophil-derived oxidants and proteolytic enzymes that disrupt the BBB and injure the brain tissue. Accordingly, the FSS-regulated integrity of BBB is seriously influenced by inflammation. However, it is still obscure how FSS influences the occurrence of inflammation in the process of ischemia and reperfusion.

EndMT is a purposeful or adaptive differentiation of endothelial cells in response to external stimuli, accompanied by the loss of cell-cell junctions and cell polarity. Multiple studies identified that FSS can effectively induce the occurrence of EndMT in vascular endothelial cells.^42^ Dal-Bianco *et al.*^7^ discovered that mechanical stretching of mitral valve leaflets in sheep mimicked the stretching effect caused by an enlarged left ventricle after myocardial infarction, leading to EndMT. Our previous results also indicated that FSS promoted the occurrence of epithelial-mesenchymal transition (EMT) and enhanced metastasis in hepatocellular carcinoma.^18^ In this study, we also found that changed FSS regulated the occurrence of EndMT in rBMECs (Fig. 5). Importantly, the phenotype transition was influenced by both the intensity and duration of FSS.

Emerging evidence suggested that endothelial cells with mesenchymal phenotype played a central role in inflammation and endothelial dysfunction.^43^ However, there is no consensus on whether EndMT promotes or hinders the progression of inflammation in cerebral IRI. On the one hand, some studies have shown that TGF-β can bind to integrins αvβ1/6 and phosphorylate specific serine residues in Smad2 and Smad3 proteins, leading to the occurrence of EndMT.^44^ On the other hand, mesenchymal endothelial cells were identified as highly pro-inflammatory cells and expressed high levels of ICAM-1, VCAM-1, and various cytokines and growth factors, eventually leading to aggravation of inflammation, and endothelial cell dysfunction.^43,45^ Therefore, we further explored the effect of EndMT in FSS-modulated inflammation during the process of ischemia and reperfusion. The *in vivo* results revealed that EndMT occurred in cerebral microvascular endothelial cells at the early stage of reperfusion, while the continuous PFSS may result in a reversal MEndT event (Fig. 5). In addition, *in vitro* results also showed that the sudden recovery of FSS at the early stage of reperfusion led to the occurrence of EndMT in rBMECs and aggravated inflammation, resulting in increased BBB permeability, and the opening of TJs. Conversely, at the end of reperfusion, with the restoration of endothelial phenotype, inflammation and BBB permeability is reduced, and TJs-associated proteins are reversed (Figs. 2 and 3). According to these results, we confirmed that altered FSS-induced EndMT aggravated inflammation and impaired BBB function during ischemia–reperfusion.

YAP is a transcriptional coregulator that can act as an intracellular mechanoreceptor protein involved in triggering EMT events in tumors.^18,46^ The Hippo/YAP signaling pathway has been shown to exert function on the BBB breakdown during cerebral IRI.^47^ However, YAP involved in FSS-induced EndMT in cerebral IRI has not been reported yet. We found that the expression of YAP was elevated in the early stage of IS in rats after middle cerebral artery embolization and was accumulated in the nucleus. At the late stage of ischemia–reperfusion, the expression of YAP in the nucleus was significantly reduced (Fig. 6). Meanwhile, the knockdown of YAP revealed that rBMECs maintained an endothelial phenotype with concomitant loss of mesenchymal phenotype and reduced inflammation during the early phase of ischemia–reperfusion (Fig. 7).

To further investigate YAP in response to FSS, the study identified the cytoskeletal signaling pathway involved in regulating the IRI process by second-generation mRNA sequencing (Fig. 4). Loading FSS after treatment of rBMECs with the cytoskeleton inhibitor Latrunculin B revealed that inhibition of the cytoskeleton resulted in fewer nuclear translocations of YAP at the early stage of reperfusion and that rBMECs regained their endothelial phenotype with less inflammation. Meanwhile, Rho GTPase was significantly upregulated during the ischemic phase (LSS groups) in response to FSS-regulated F-actin-mediated YAP translocation to the nucleus for activation, and expression was significantly downregulated during the early reperfusion phase (LSS/EP groups), demonstrating that the occurrence of EndMT in rBMECs is dependent on Rho GTPases (Fig. S5).

**FIG. 9.**
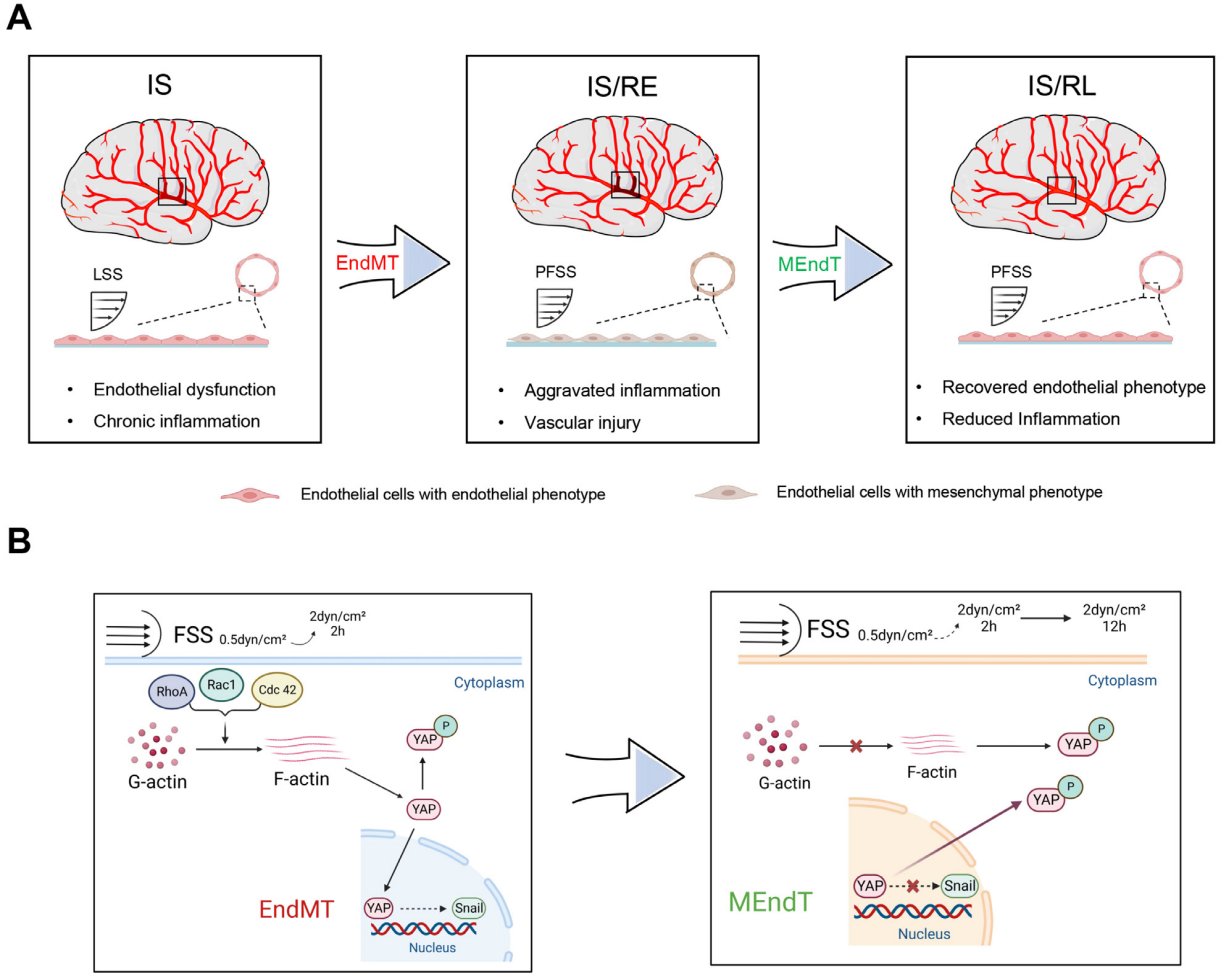
Schematic of FSS-induced endothelial phenotypic transition resulting in endothelial inflammation that contributed to ischemia–reperfusion injury and repair. (a) At early reperfusion, restored blood supply (increased strength of shear stress) induced an EndMT event in Ecs, resulting in aggravated inflammation and vascular injury. At late reperfusion, ECs gradually adapt to physiological shear level (increased duration of shear stress), recover endothelial phenotype (MEndT), and reduce endothelial inflammation. (b) Exposed to FSS with continuous change in strength and duration, ECs experienced an endothelial-mesenchymal phenotype (from EndMT to MEndT), which is dependent on Rho GTPases—F-actin to regulate the activation of YAP. IS ischemia; IS/RE: ischemia–reperfusion at an early stage; IS/RL: ischemia–reperfusion at a late stage; LSS: low fluid shear stress; NFSS: normal physiological fluid shear stress.

Taken together, we demonstrated that FSS, changed from LSS to PFSS in the early reperfusion, induced inflammatory injury and brain damage via regulating EndMT transformation in rBMECs. However, with the increased duration of reperfusion, endothelial cells gradually adapt to the PFSS and experience a reversal MEndT event, thereby restoring their endothelial phenotype and alleviating both endothelial inflammation and brain damage during late reperfusion. The schematic representation of these findings is depicted in Fig. 9.

## IV. CONCLUSION

In the early phase of reperfusion, endothelial cells exposed from LSS to PFSS induced a phenotypic transition known as EndMT, leading to aggravated endothelial inflammation and brain damage. In the later stage of reperfusion, endothelial cells gradually adapted to the physiological level of FSS, and experienced a reversal MEndT event in a time-dependent way that contributed to the endothelial repair of ischemia–reperfusion [Fig. 9(a)]. YAP plays a role in mediating FSS-induced EndMT during the early stages of IRI through the Rho GTPases-F-actin signaling pathway. Sustained FSS following reperfusion may lead to YAP phosphorylation retained in the cytoplasm, thus triggering MEndT events that contribute to endothelial repair of ischemia–reperfusion [Fig. 9(b)].

## V. METHODS

### A. Rat MCAO model

All animal works were performed with the approval of the Medical Ethics Committee of Sichuan University (K2021015). All ethical guidelines for experimental animals were followed. A total of 48 adult male Sprague–Dawley (SD) rats, weighting 200–220 g, were randomly divided into four groups (n = 12/group), including Sham (sham operation groups), IS (ischemia 2 h), IS/RE (ischemia 2 h + reperfusion 1d), and IS/RL (ischemia 2 h + reperfusion 30d) [Fig. S1(a)]. The MCAO model was performed as previously described.^48,49^ Briefly, the rat was anesthetized with a mixture (2%–2.5% isoflurane and 97.5%–98% air) and fixed supine on a 36 °C heating pad. Cut along the midline of the neck and expose the right common carotid artery (CCA), the external carotid artery (ECA), and the internal carotid artery (ICA). The ECA was ligated, and a vessel clip was placed on the CCA and another on the ICA. A small cut was made on the ECA and a nylon thread with a silicone tip (5–6 mm in length, 0.33–0.35 mm in diameter, MSRC35B200PK50, RWD Life Science Co, Ltd) was inserted into the ECA. The vessel clip on the ICA was removed, and the nylon thread advanced 18–19 mm until resistance was felt. After 2 h of occlusion, the nylon thread was removed and the wound was sutured. The sham operation group was also given anesthesia and an incision in the middle of the neck, then the skin was sutured, and a normal diet was resumed. The MCAO models were assessed by laser speckle photometry (RFLSI III) according to the middle cerebral artery blood flow (CBF) index. The neurobehavioral changes were evaluated in each group using the Zea-longa scoring system.^50^ Briefly, 0 points for no nerve damage; 1 point for mild nerve damage, with the contralateral forelimb adduction during the tail lift; 2 points for moderate nerve damage, with contralateral rotation when crawling; 3 points for severe nerve damage, standing or rotation to the opposite side when crawling; 4 points for involuntary movement with disturbance of consciousness; and 5 points for death. Those with a score of 1–3 are considered successful models.

### B. Cell culture

The rat brain microvascular endothelial cells (rBMECs) were purchased from Guangzhou Jennio Biotech Co, Ltd. The cells were cultured with complete RPMI-1640 growth medium (Gibco, Invitrogen), containing 10% fetal bovine serum (FBS, Shanghai Zhongqiao Xinzhou Biotech Co, Ltd.), 100 units/ml and 100 *μ*g/ml penicillin–streptomycin at 37 °C in an incubator with 95% humidified air and 5% carbon dioxide (Heraeus Company, Germany).

### C. FSS loading

In this study, the rBMECs with 90% confluence were seeded on a glass slide which was immediately placed in parallel plate flow chambers and exposed to a stable FSS. The FSS was calculated as previously described.^51^ Briefly, the loading shear stress was calculated by the following formula:

$$\tau =6\frac{\mu Q}{{WH}^{2}},$$

where τ is the FSS loaded to the cells; *μ* is the viscosity of the circulating buffer (0.83 mPa s); Q is the flow rate; H (0.3 mm) is the height of the chamber, and W (74.80 mm) is the width of the chamber.

Normal PFSS of rat cerebral microvascular ranged from 1 to 6 dyn/cm^2^.^30^ Accordingly, 0.5 and 2 dyn/cm^2^ FSS were applied as the LSS and PFSS, respectively. The rBMECs were exposed to LSS of 0.5 dyn/cm^2^ (ischemia period, LSS groups) for 6 h and subsequently recovered to the normal physiological level of 2 dyn/cm^2^ for 2 h (early stage of ischemia–reperfusion, LSS/EP groups) and 12 h (late stage of ischemia–reperfusion, LSS/LP groups), respectively [Fig. S1(b)]. As controls, the cells were cultured at static conditions (Ctrl groups) and exposed to a normal physiological level of 2 dyn/cm^2^ (PFSS groups), respectively.

### D. 2,3,5-triphenyl tetrazolium chloride (TTC) staining

At 0 h, 24 h, and 30 d reperfusion after 2 h ischemia, the brain tissues were taken out once the rats were anesthetized by a mixture of 2%–2.5% isoflurane and 97.5%–98% air (three rats in each group) for further TTC staining. The steps are as follows: First cut off the head and trim off the muscles around the occipital bone, then pry open the occipital bone and parietal bone and lift the brain from the front. The brains were cut into six coronal slices of 2 mm and soaked in 2% TTC with no light sheltered for 30 min at 37 °C. We took pictures of each slice and analyzed the infarct size with image J. The cerebral ischemic volume was calculated by multiplying the area by the thickness (2 mm). To reduce the effects of edema, the corrected infarct volume was equal to the contralateral hemisphere volume minus the non-infarct volume measured in the ipsilateral hemisphere, as previously described.^52,53^

### E. Evaluation of blood–brain barrier (BBB) leakage by Evans blue staining

BBB permeability was assessed by Evans blue (EB) staining. Rats were anesthetized with a mixture of 2%–2.5% isoflurane and 97.5%–98% air and EB (2%, 4 ml/kg, normal saline, Solarbio) was injected into the tail vein of the stroke rat. After the EB cycle for 1 h, the left ventricle was slowly perfused with normal saline to wash out the EB. The brain tissue was cut into five pieces and photographed quickly. Left hemispheres were isolated and homogenized with 50% trichloroacetic acid (1.5 ml/g, Solarbio) for 1 min to take immune fluorescence. The tissue was kept at 4 °C overnight, centrifuged at 14 000 rpm for 15 min, and the supernatant was collected. The fluorescence intensity was detected by a full-wavelength microplate reader (excitation at 620 nm and emission at 680 nm). Data are represented as concentration of EB dye (*μ*g)/tissue weight (g).

### F. Magnetic resonance imaging (MRI) measurements

The infarct volume of the brain in four groups (n = 3/group) was detected by a 7 T small animal nuclear magnetic resonance system (Bruker Bio Spin, Germany). The rat was anesthetized by air mixture with 2%–2.5% isoflurane and 97.5%–80% air and MRI-compatible equipment was used to monitor the body temperature and respiratory rate of the rats. High-resolution T2-weighted images (hrT2wi) were acquired on a 7 T MR scanner (Bruker Bio Spin, Ettlingen, Germany) and a 20 mm inner diameter transmit/receive volume coil (RAPID Biomedical, Rimpar, Germany) with fast spin echoes, TR\TE = 2500\33 ms, voxel size = 0.1 × 0.1 × 0.3 mm^3^, turbo factor = 7, and slice thickness = 2 mm. The planar directions are referred to as x (lateral) and y (ventral–dorsal), while the slice selection direction z refers to the caudal–rostral axis.

### G. Transmission electron microscope (TEM)

Brains in each group (n = 3) were collected. Tissues from midbrain ischemic sites with a size of 1 × 1 × 1 mm^2^ were taken and fixed in 3% glutaraldehyde for 24 h. After successively passing through 1% OsO_4_ at 4 °C, ethanol and acetone were fractionally dehydrated, embedded in a 1:1 mixture of Epon812 and acetone, placed at 37 °C for 24 h and 60 °C for 48 h, and finally used ultramicrotome sections (about 60 nm thick) and double-stained with uranyl acetate and lead citrate. Tight junctions were observed with TEM. In addition, rBMECs in each group were collected and centrifuged at 1500 rpm for 15 min. The supernatant was discarded 0.5% glutaraldehyde fixative was added, and 3% glutaraldehyde fixative was added after standing at 4 °C for 8 min. Then, the samples were fixed in 1% osmium tetroxide, dehydrated in acetone, embedded in Epon812, ultrathin sectioned, and double-stained with uranyl acetate and lead citrate. The Weibel-Palade body and tight junctions were observed with TEM.

### H. Immunofluorescence

Immunofluorescence staining was performed as previously described.^54^ Briefly, brains were sliced into 10 *μ*m thick coronal sections (Bregma: –100 to +100 *μ*m) and fixed with 4% paraformaldehyde for 30 min at room temperature. Then, it was permeated with 0.25% Triton X-100 for 15 min and blocked with 1% goat serum for 2 h. After blocking, brain slices were incubated with a primary antibody mixture at 4 °C overnight, followed by appropriate secondary antibodies (1:1000, 1% goat serum/PBS) for 2 h at room temperature. Nuclei were stained with 4,6-diamino-2-phenyl indole (DAPI; 1:800) for 10 min. Images were acquired by confocal microscopy (Zeiss, LSM710).

In addition, rBMECs were fixed with 4% paraformaldehyde for 20 min, permeated the cell membrane with 0.5% Triton X-100 for 10 min, and blocked with 1% goat serum for 30 min. After blocking, cells were incubated with a primary antibody mixture at 4 °C overnight, followed by appropriate secondary antibodies (1:1000, 1% goat serum/PBS) for 2 h at room temperature. Nuclei were stained with 4,6-diamino-2-phenyl indole (DAPI; 1:800) for 10 min. Images were acquired by confocal microscopy (Zeiss, LSM710). As an inhibitor of actin polymerization, Latrunculins B (Lat B) could effectively sequester monomeric G-actin, leading to extensive disassembly of the F-actin. Therefore, Lat B was employed to suppress actin polymerization and investigate the dependence of YAP-mediated EndMT on F-actin.

### I. F-actin staining

rBMECs were fixed with 4% paraformaldehyde for 20 min, permeated the cell membrane with 0.5% Triton X-100 for 10 min, and blocked with 1% goat serum for 30 min. After blocking, it was incubated with fluorescent phalloidin solution (Texas Red-X phalloidin, 1:200, Invitrogen Company) for 30 min and with DAPI (1:800) for 10 min. Images were acquired by confocal microscopy (Zeiss, LSM710).

### J. Western blot (WB) analysis

The total protein in each cell group was extracted with cell lysis buffer and then centrifuged at 14000 rpm for 10 min at 4 °C, and the supernatant was taken. The protein concentration of the sample was determined using a bicinchoninic acid (BCA) protein assay kit. Then, the protein was denatured, and an equal amount (25 *μ*g) of protein in each group was added to 10% or 12% SDS-PAGE gel for electrophoresis and transferred to the PVDF membrane. After blocking in 5% BSA at room temperature for 2 h, the membrane was incubated with primary antibody overnight at 4 °C. The next day, the membrane was washed (3 × 5 min) with TBST and incubated with the corresponding HRP-conjugated secondary antibody at room temperature for 2 h. Subsequently, after washing (3 × 5 min) with TBST, the membrane was detected using the^®^ ChemiDoc™ XRS+ system and Image Lab™ software. GAPDH was used as an internal reference for quantitative analysis. The antibodies of inflammation, EndMT & MEndT, and YAP-related markers were used for WB analysis and immunofluorescence staining. The details of the antibodies are shown in the supplementary material in Table S1.

### K. Quantitative RT-PCR

The total RNA of each group was extracted according to the operating instructions of the Cell and Animal Total RNA Isolation Kit (Foregene, China), and Nanodrop 2000 (Thermo Fisher Company, USA) was used to quantify the total RNA. The total RNA was then reverse transcribed into cDNA at the cellular and animal levels according to the instructions of the gDNA Eraser (Perfect Real Time, Takara, Japan) PrimeScript^®^ RT kit. Then, using SYBR^®^Premix Ex TaqII (Tli RNaseH Plus), 40 cycles of 3 min at 94 °C, 10 s at 94 °C, 60 s at 60 °C, and 30 s at 72 °C were performed in sequence to realize the polymerase chain reaction (PCR). GAPDH as the internal reference gene and the relative expression of the target gene was calculated by 2^–△△Ct^ analysis. Each experiment was repeated three times according to the original plan to end the experiment. Gene primers were designed and synthesized by Sangon Biotech Co., Ltd. (Sangon Biotech, China), as shown in the supplementary material (Table S2).

### L. RNA-sequence

The rBMECs were collected from LSS groups, LSS/EP groups, and LSS/LP groups, respectively. Total RNA was extracted from the sample by using the TRIzol reagent (Invitrogen Company, USA). Before preparing the sequencing library by total RNA, quality control was determined by using agarose gel electrophoresis, and quantification was checked by Nanodrop 2000 (Thermo Scientific). Agilent 2100 was used for controlling library quality, which was quantified by qPCR. Then, Illumina Hiseq 4000 was applied for sequencing. Using FastQC software to detect sequence quality, the abundance of transcripts in each sample was estimated by String Tie, and the FPKM value (≥0.5) of genes and transcripts was assessed using the R package Ballgown. Subsequently, R package Ballgown was used to filter differentially expressed genes and transcripts. Based on gene expression level (principal component analysis, PCA), correlation analysis was processed. For hierarchical clustering, gene ontology (GO), and pathway analysis, only genes with a change in 1.5-fold or greater were considered. The whole data have been uploaded to the GEO repository (NCBI tracking system No. 24206802).

### M. Transfected DNA plasmids

The recombinant plasmid was constructed by ligating a shRNA gene fragment targeting the YAP sequence to the pGPU6/GFP/Neo vehicle using RNA interference technology. The shYAP gene sequence is as follows: CTGGTCAGAGATACTTCTTAA. The recombinant plasmids were transfected into rBMECs using Lipo8000™ transfection reagent. The transfection effects were observed under an inverted fluorescence microscope after 24 h incubation in a cell incubator. The successfully transfected rBMECs can produce green fluorescent protein.

### N. Statistical analysis

GraphPad Prism 6 (La Jolla) was used for data analysis. All data were represented as mean ± standard deviation (SD). The difference between groups was compared by using one-way ANOVA and Tukey's test. *P* < 0.05 was considered a significant difference.

## SUPPLEMENTARY MATERIAL

See the supplementary material for the details of *in vivo* and *in vitro* experimental groups shown in Fig. S1 and the laser speckle images and analyses during ischemia and reperfusion given in Fig. S2; the changes in mesenchymal markers in cerebral vessels during ischemia and reperfusion and the expressions of MMP2 and α-SMA are shown in Figs. S3 and S4, respectively; the expression of Rho GTPases in rBMECs and the distribution and expression of endogenic TNF-α in endothelial cells exposed to FSS were given in Figs. S5 and S6, respectively; and for other primer sequences for qRT-PCR detection and detailed information on antibodies, see Tables S1 and S2.

## Data Availability

The data that support the findings of this study are available within the article and its supplementary material.
